# Polyvinylamine Membranes Containing Graphene-Based Nanofillers for Carbon Capture Applications

**DOI:** 10.3390/membranes9090119

**Published:** 2019-09-12

**Authors:** Riccardo Casadei, Davide Venturi, Marco Giacinti Baschetti, Loris Giorgini, Emanuele Maccaferri, Simone Ligi

**Affiliations:** 1 Department of Civil, Chemical, Environmental and Material Engineering (DICAM), University of Bologna, Via Terracini 28, 40131 Bologna, Italy; riccardo.casadei11@unibo.it (R.C.); davide.venturi16@unibo.it (D.V.); 2 Department of Industrial Chemistry “Toso Montanari”, University of Bologna, Viale del Risorgimento 4, 40136 Bologna, Italy; loris.giorgini@unibo.it (L.G.); emanuele.maccaferri3@unibo.it (E.M.); 3 Graphene-XT S.r.l., Via Massimo d′Azeglio 15, 40131 Bologna, Italy; simone.ligi@graphene-xt.com

**Keywords:** nano-composite membranes, facilitated transport, carbon capture, graphene, graphene oxide

## Abstract

In the present study, the separation performance of new self-standing polyvinylamine (PVAm) membranes loaded with few-layer graphene (G) and graphene oxide (GO) was evaluated, in view of their use in carbon capture applications. PVAm, provided by BASF as commercial product named Lupamin^TM^, was purified obtaining PVAm films with two degrees of purification: Low Grade (PVAm-LG) and High Grade (PVAm-HG). These two-grade purified PVAm were loaded with 3 wt% of graphene and graphene oxide to improve mechanical stability: indeed, pristine tested materials proved to be brittle when dry, while highly susceptible to swelling in humid conditions. Purification performances were assessed through FTIR-ATR spectroscopy, DSC and TGA analysis, which were carried out to characterize the pristine polymer and its nanocomposites. In addition, the membranes′ fracture surfaces were observed through SEM analysis to evaluate the degree of dispersion. Water sorption and gas permeation tests were performed at 35 °C at different relative humidity (RH), ranging from 50% to 95%. Overall, composite membranes showed improved mechanical stability at high humidity, and higher glass transition temperature (T_g_) with respect to neat PVAm. Ideal CO_2_/N_2_ selectivity up to 80 was measured, paired with a CO_2_ permeability of 70 Barrer. The membranes’ increased mechanical stability against swelling, even at high RH, without the need of any crosslinking, represents an interesting result in view of possible further development of new types of facilitated transport composite membranes.

## 1. Introduction

In last decades, the fast economic growth of both industrialized and developing countries has brought a significant increase of global energy demand, which has been usually fulfilled by burning fossil fuels. Consequently, combustion products, such as CO_2_ and other greenhouse gases [1], have been accumulated in the atmosphere: from 1850 to 2017, the average CO_2_ concentration has risen from 280 to 400 ppm, determining the well-known global temperature increase [2,3]. 

A shift towards renewable-based energy sources is considered the best long-term solution to reduce this problem, but it represents a long and difficult process. Thus, more immediate actions are needed to efficiently tackle global warming [4]. In this context, a mid-term solution can be represented by Carbon Capture and Storage (CCS) technologies, which focus on the efficient recovery of carbon dioxide prior to its emission and on its storage in suitable location, in order to prevent its leakage in atmosphere [5].

Many CCS technologies have been studied [6], for both pre- and post-combustion applications, such as solvent absorption [7], adsorption on porous supports [8], gas membrane separation [9,10], mineralization [11,12] and cryogenic separation [13]. Among these, gas membrane separation is showing promising results as an upcoming technology thanks to a series of advantages such as low installation and management costs, reduced energy requirements and lower environmental impact, due to the absence of chemical solvents, compared to others technologies [9,14,15]. Besides these advantages, membrane separation techniques also possess some limitations. The main performance parameters, selectivity and permeability, are often not independent from each other: usually, membranes with high permeability show low selectivity for a given gas couple and vice versa. This kind of behavior is well shown by Robeson’s upper bound [16], which graphically represents this permselectivity trade-off, considering most of the experimental data published in the open literature. For this reason, it is also regarded as the state of the art for gas separation membranes and is often used to evaluate the performances of new membrane materials.

In post combustion CCS, CO_2_ permeability and CO_2_/N_2_ selectivity are the most critical parameters and, to reach both high-quality separation and performance above the upper bound, several approaches have been used, from the use of composite materials and mixed matrix membranes [17,18,19,20,21], ionomer and ionic liquids with high CO_2_ affinity [22,23,24,25], to the use of Facilitated Transport Membranes (FTMs) [26,27,28,29,30,31,32,33].

The latter membranes, in particular, rely on reversible chemical reactions between the target gases and specific functional groups (*carriers*) embedded in the membrane’s matrix. This way, only the reacting species are transported across the membrane by this mechanism, increasing theoretically both permeability and selectivity. In addition, facilitated transport allows the separation processes to be conducted with a lower pressure gradient, resulting in further reduction of operative costs [26,34,35]. Carriers in FTMs can be both *mobile* or *fixed:* mobile carriers are chemical species free to move within the matrix, which can bound the target molecule at the feed side, transport it across the membrane and then release it at the permeate side. Fixed carriers are instead represented by the reactive sites linked to the matrix and endowed with a limited degree of freedom, which can be used by the reacting species to “hop” from one side of the film to the other.

The possible use of basic carriers to exploit CO_2_ acidic character encouraged many groups to use this approach in gas separation membranes, achieving interesting results with the use of amine based carriers [27,29,36,37,38,39,40,41,42].

**Figure 1:**
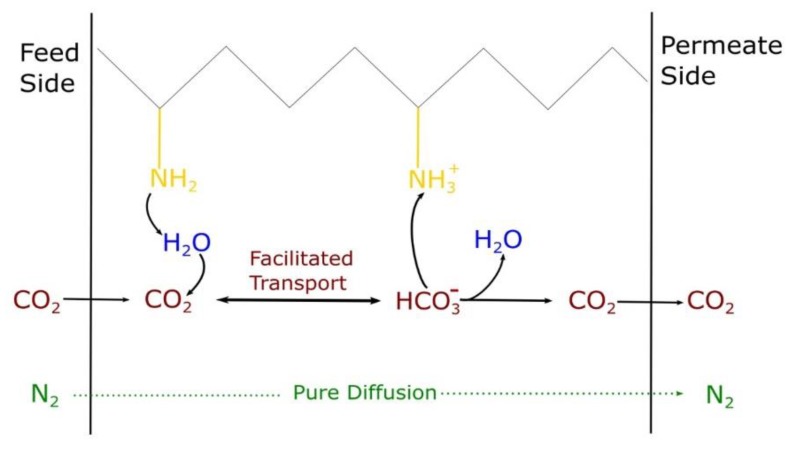
Example of Facilitated Transport Membranes (FTM) mechanism.

Amines are indeed able to bound CO_2_ selectively through different possible chemical mechanisms, such as the ones shown in Equations (1)–(5). Among the many different available materials, polyvinylamine (PVAm), which has a high density of primary amine groups, as schematized in Figure 1, has often been considered as a convenient medium to selectively transport carbon dioxide [9,26].

CO_2_ + H_2_O → H_2_CO_3_ (1)

H_2_CO_3_ + R-NH_2_ → HCO_3_^−^ + R-NH3^+^ (2)

HCO_3_^−^ + R-NH_3_^+^ → H_2_CO_3_ + R-NH_2_ (3)

H_2_CO_3_ → CO_2_ + H_2_O (4)

Simplifying:

CO_2_ + H_2_O + R-NH_2_ ⇄ HCO_3_^−^ + R-NH_3_^+^ (5)

As shown in Equations (1)–(5) and Figure 1, the CO_2_ facilitated transport mechanism requires water to occur, therefore FTMs need to work in the presence of humidity.

Another important parameter to consider is pH, as it influences amine protonation equilibrium (Equations (6) and (7)), which in turn affects CO_2_ interaction with the active carrier:

R-NH_2_ + H^+^ → R-NH_3_^+^ (6)

R-NH_3_^+^ + OH^−^ → R-NH_2_ + H_2_O (7)

Thus, to exploit the facilitated transport mechanism presented in Equations (1)–(5), neutral amine form is needed and it is recommended to maintain high pH values during material purification and membrane preparation, as also demonstrated by Kim et al. [43].

Unfortunately, one of the main disadvantages of PVAm is its mechanical weakness, especially at high water content, where the self-standing PVAm membranes tend to swell and break, thus it is very difficult to use them for FTMs application in pure form. For this reason, many strategies have been developed to increase PVAm mechanical resistance, thus creating more stable membranes. Crosslinking the matrix [27,44], using polymer with higher molecular weight [45] and adding nano-materials [39,46] to obtain composite and mixed matrix membranes (MMM) are among the solutions considered.

Following the latter idea, this study investigated the use of graphene-based nanofillers in PVAm to limit membranes′ swelling and failure in highly humid environmental conditions. Few layer graphene (G) and graphene oxide (GO) were chosen for their great mechanical properties and well known reinforcing behavior [47,48,49,50]. In addition, these sheet-like graphenic nanofillers have also been reported to be very good building blocks for mixed matrix membranes able to influence permeation properties such as selectivity and permeability [51,52,53,54]. In some cases, graphene-based MMM resulted in being able to reach extremely high values of permeances and selectivity, beyond those usually requested for post combustion CCS applications [55], making these nanomaterials interesting candidates also for a further improvement of PVAm based membrane performances. 

In this preliminary activity, the main purpose was to obtain mechanically stable films even at high humidity conditions and to determine the effect of the graphene-based reinforcements, in terms of both materials structure and gas separation performances.

Different membrane samples containing 3 wt% of graphenic derivatives were produced and studied through FTIR-ATR, TGA, DSC and SEM techniques before carrying out water vapor uptake and CO_2_/N_2_ permeation experiments at 35 °C with relative humidity ranging from 50% to 95%.

## 2. Materials and Methods 

**Figure 2:**
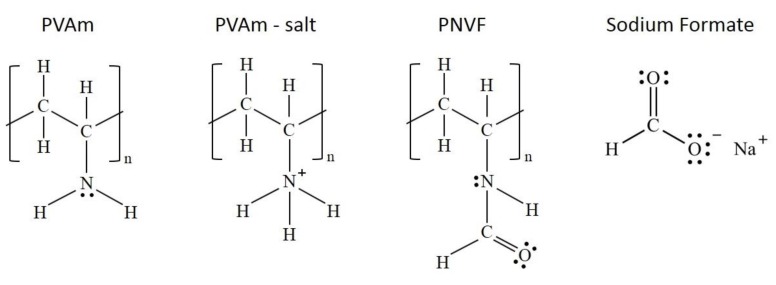
Components of commercial PVAm solution containing 5% molar ratio of PNVF with respect to PVAm and PVAm salt.

Polyvinylamine (PVAm) for membrane preparation was sourced from the commercial product Lupamin™ 9095, kindly provided by BASF Italia S.p.A. (Cesano Maderno, Italy) in a water solution with a reported total solid concentration of 20–22 wt% and pH = 7–9. The dissolved solids consist of a copolymer poly(vinylamine-co-N-vinyl formamide) (PVAm-co-PNVF) (95% degree of hydrolysis) with a reported molecular weight of 340,000 Da and salts, mainly sodium formate, a byproduct of PVAm′s synthesis [56], and sodium chloride. Figure 2 summarizes the different components in the commercial solution, which is referred to from now on as Lupamin 9095.

Graphene (G) and graphene oxide (GO) were kindly provided by Graphene-XT S.r.l: the former was supplied in powder form with platelets of 5 micron lateral dimension and 2–8 nm thickness, while the latter, produced by a modified Hummers’ method, was supplied in water solution containing over 90% monolayer sheets with a solid concentration of 2 mg/g, an oxidation degree around 40% and an average lateral sheets dimension of 20 micron.

Both nanofillers presented a “few-layer” structure [57,58], with an average particle dimension of 5 µm × 5 µm × 6–8 nm for graphene and 50 µm × 80 µm × 1 nm for graphene oxide.

All polymeric solutions were prepared in deionized water (conductivity 12 µS/cm). During the purification process, 96% ethanol (Sigma-Aldrich, St. Louis, MO, United States) and a strong anionic exchange resin (Amberlite IRA958Cl, Dow Chemical, Midland, MI, USA), were employed.

### 2.1. Purification

**Figure 3:**
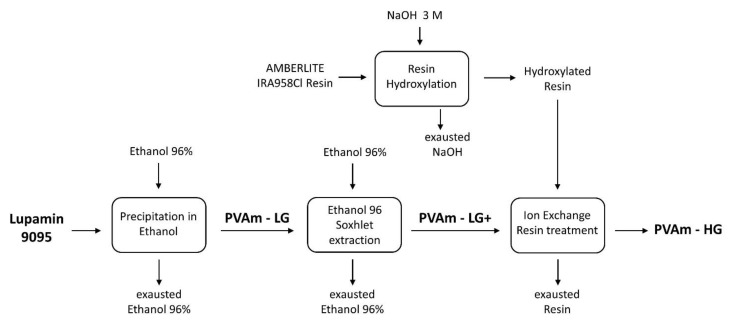
Scheme adopted for commercial Lupamin 9095 purification.

The commercial form, Lupamin 9095, contains a relatively high amount of salt, which negatively influences the membrane’s structure, making it extremely heterogeneous and unusable for required applications. Thus, to achieve an acceptable degree of purification, a multi-step protocol was implemented, as schematized in Figure 3, partially following the procedure previously reported by Ho et al. [29].

Firstly, PVAm present in Lupamin 9095 solution was precipitated by adding ethanol to the polymeric solution to reach a 4:1 alcohol/solution volume ratio; the precipitated fraction of polymer was then recovered, dried in oven at 60 °C overnight and stored with the label of PVAm-LG (Low Grade).

Subsequently, to further reduce salt impurities, the dried polymer was finely cut into small particles, to maximize the surface area, and subjected to Soxhlet extraction in 96% ethanol, for 48 h, obtaining the so-called PVAm-LG+.

As a final step, after a second drying stage at 60 °C, the polymer obtained was dissolved in water with a concentration of 2.5 wt% and the ion exchange resin was added to obtain a slurry with a 1:15 polymer content to resin mass ratio. Prior to the addition, the resin was hydroxylated by treating it with a 3 M NaOH solution. Ion exchange was carried out under continuous magnetic stirring and periodically measuring the solution’s pH until no variation was observed, indicating the end of ion transfer. The final pH value measured was always between 11.9 and 12.0, obtained after 2 h of stirring.

Finally, vacuum filtration was performed, to separate the resin from the purified polymeric solution, which was once again dried at 60 °C and named PVAm-HG (High Grade).

### 2.2. Membrane Preparation

All membranes prepared and analyzed were self-standing films, obtained by solvent casting technique. Both pure and graphene loaded films were prepared for comparison. 

Pristine PVAm films were prepared by pouring a polymeric aqueous solution with a concentration equal to 2.5 wt% in a PTFE Petri dish. The solution was covered and then dried at 60 °C in air for two days.

For the nano-composites films, the compounding process was carried out by adding a given amount of filler (in powder or suspension form) to the solution to achieve a graphene concentration with respect to the polymer content of 3 wt%, as suggested by nanofiller producer. To ensure a proper dispersion, the suspension obtained was stirred at 800 rpm via magnetic stirring for 30 min and then immersed in a sonication bath (AVO ST-3, LAVO s.r.l., Giussano, Italy) at 50–60 Hz, a treatment which can also decrease sheet’s dimensions [59]. For unmodified graphene, 30 min of sonication were enough to achieve a reasonable dispersion, while for graphene oxide up to 2 h were needed. As a final mixing step, the whole mixture was passed twice through a three-roll mill (Exakt 35/50, Norderstedt, Germany), achieving a total residence time of one minute.

Once the nano-composite solution was obtained, the films were casted as previously described for the pristine PVAm.

The thickness of the different films was measured by using a flat plate micrometer (Digital Absolute Micrometer Series 227-22, Sakado, Japan) and each film resulted to be in the range of 60–120 µm; maximum variation of thickness in each sample never exceeded 5%.

### 2.3. Chemical-Physical Characterization 

Chemical-physical characterization was carried out on dry samples to verify the efficiency of purification methods and the proper dispersion of the nanofiller. Additional tests, made to obtain a preliminary estimation of the effect of nanofiller on other materials properties, were also conducted and are here reported for the sake of completeness, even if they have limited interest for the analysis of the membrane permeation properties. To favor the facilitated transport mechanism, membranes are usually tested at high humidity and thus in a water swollen state, which presents a completely different structure with respect to the dry polymer.

#### 2.3.1. FTIR-ATR

The main technique used for evaluating the extent of the purification was the Fourier Transform Infrared Spectroscopy, which allowed verifying the removal of impurities and assess the presence of the desired functional groups.

The apparatus used was a FT–IR Nicolet 380 (Thermo Fisher Scientific, Waltham, Massachusetts, United States) endowed with a single bounce diamond crystal ATR base (MIRacle™ Single Reflection, Pike Technologies, Madison, WI, United States).

To ensure complete adhesion, the polymeric samples were casted directly on the crystal, using a hot air flow, at about 50 °C, to speed up the drying step.

Recorded spectra (32 scans per analysis, resolution of 4 cm^−1^) were analyzed through the Omnic software package.

#### 2.3.2. TGA

Thermal and thermo-oxidative degradation of commercial Lupamin 9095 and purified PVAm membranes was evaluated through thermogravimetric analysis (TGA, Q600, TA Instruments, New Castle, England), coupled with an infrared spectrometer (Cary 660, Agilent Technology, Santa Clara, CA, United States).

Samples were preventively dehydrated by heating at 60 °C in air for 24 h and subsequently vacuum dried for 6 h at room temperature to minimize the water content. Samples were tested with the following program: heat from room temperature (about 20 °C) to 600 °C at a rate of 10 °C/min in nitrogen atmosphere, 5 min isotherm, switch to atmospheric air, and final isotherm of 30 min to oxidize organic residues.

The evolved gas during TGA analysis was analyzed qualitatively through IR spectrometry (recorded 6 spectra per minute, resolution of 8 cm^−1^).

#### 2.3.3. DSC

Differential Scanning Calorimetry (DSC) was adopted to investigate thermally induced phenomena in the polymers. DSC measurements were carried out on a DSC Q2000 (TA Instruments, New Castle, England). Samples (10 mg) were heated from 0 to 130 °C, cooled to 0 °C, and then heated to 150 °C (heating/cooling rate 10°C/min) in nitrogen atmosphere. 

It was decided to not heat over 150 °C to avoid abnormal signals and distorted results, which could be caused by membrane degradation. Indeed, reports exist indicating for PVAm degradation temperatures as low as 140 °C [60].

#### 2.3.4. SEM

Scanning Electron Microscopy (SEM) was used to evaluate the nano-reinforcement dispersion and the aspect of the nano-modified composite, besides the presence of impurities. The apparatus used was an EVO 50EP from Zeiss (Oberkochen, Germany) provided with an Everhart–Thornley detector, using a focused beam of electrons at 20 kV and magnification of 500×–1000×. Samples were prepared by fragile fracture in liquid nitrogen in order to observe the cross section.

### 2.4. Mass Transport Properties

#### 2.4.1. Water Sorption

To determine the water uptake of the cast films, a quartz spring microbalance was used [61,62]; tests were carried out in the range of 15–90% RH, at 35 °C.

Within this setup, a film sample of known weight and thickness was connected to the end of a quartz spring, which was then placed in an isolated thermostatic glass column. 

Tests were made by a consecutive multi-step procedure. Initially, vacuum was applied to the column, allowing all volatile components to desorb from the sample. Once the weight was stabilized, the column was filled with a known water pressure and the sample’s displacement was recorded in real time via a digital camera (DVT Smartimage Sensor 630, Cognex, Natick, MA, United States). Once equilibrium was reached, a higher vapor pressure was fed to the column, allowing another sorption step to take place.

The sample’s weight variation due to vapor uptake at each step was calculated via Equation (8):

(8) $${\left[m_{water}\right]}_{i}=\left(h_{i}-\;h_{0}\right)\cdot \;\frac{k}{g}$$

where *k* is the spring elastic constant, *h_i_* the spring length at step *i*, *h_0_* is the initial spring length, and *g* is the gravity acceleration. Buoyancy effects were neglected in calculation due to the low pressure considered in experiments.

#### 2.4.2. Gas Permeation

Membranes processes are based on the physical separation of one or more specific chemicals through a semi-permeable barrier: compounds that can cross the barrier will accumulate in the permeate flow, while the others will remain in the retentate flow. Generally, the most important parameters in this kind of separation are the permeability, *P*, and selectivity, *α_i,j_*, which are usually defined using Equations (9) and (10), respectively:

(9) $$J_{i}=\;\frac{P_{i}\;\cdot \;\Delta p_{i}}{L}$$

where *J_i_* is the molar flow per unit surface of compound *i* through the film, *L* is the membrane’s thickness, *P_i_* is the permeability and *∆p_i_* is the partial pressure gradient between upstream and downstream for the given compound.

(10) $$\alpha_{i,j}=\;\frac{y_{i}^{d}/\;y_{j}^{d}}{y_{i}^{u}\;/\;y_{j}^{u}}$$

where *α_i,j_* is the membrane’s selectivity between components *i* and *j*, and *y* represents the molar fraction of *i* and *j* in both downstream and upstream compartments of the membrane module. Selectivity is generally defined for mixed gas experiments, but can be approximated by “ideal selectivity”, which is defined as the ratio between the permeabilities of two gases obtained separately (Equation (11)):

(11) $$\alpha^{*}{}_{i,j}=\;\frac{P_{i}}{P_{j}}$$

**Figure 4:**
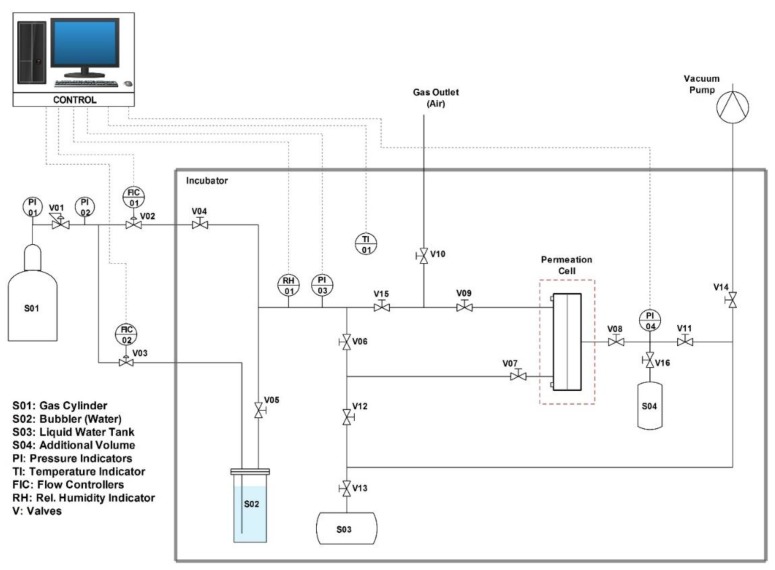
Layout of humid permeation setup.

In this work, permeability and ideal selectivity were measured through the permeation apparatus schematized in Figure 4.

This system is based on a barometric technique [63] where permeability is measured by monitoring the pressure increase, caused by the permeating gas, in the downstream volume (calibrated and constant). Hence, it can be calculated via the following equation:

(12) $$P=\;{\left(\frac{dp_{1}}{dt}\right)}_{t\to \infty }\;\frac{V}{RT}\;\frac{L}{A}\;\frac{1}{\left(p_{2}-\;p_{1}\right)}$$

where *A* represents the permeation area, *V* is the downstream volume, *T* is the system temperature, and *p_1_* and *p_2_* are, respectively, the downstream and upstream partial pressure of the considered compound.

Before every test, the membrane was placed in the permeation cell and left under vacuum overnight to remove any volatile species absorbed. Next, both the membrane and the stream to be fed to the system were equilibrated at the desired relative humidity (RH): the flow’s RH was monitored through a hygrometer (Dew Track II, Edgetech Instruments) while the membrane’s RH was controlled by the water pressure of the system, monitored via a pressure indicator (PTX 1400, Druck, Leicester, England).

When humidity levels were stabilized, the permeation test was started by exposing the upstream side of the film to the humidified feed gas. Being water in equilibrium on both sides of the membrane, the only compound eventually contributing to the pressure increment downstream was the tested gas, that is, in the present study, CO_2_ or N_2_.

## 3. Results and Discussion

### 3.1. Chemical-Physical Characterization 

#### 3.1.1. FTIR-ATR Characterization

**Figure 5:**
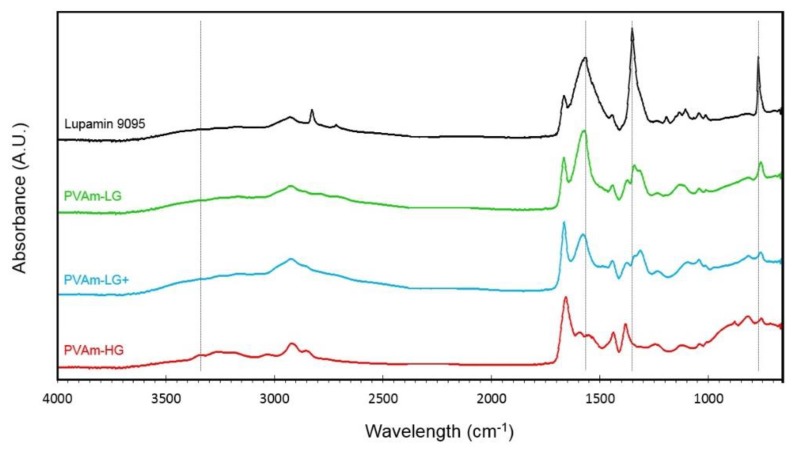
FTIR-ATR spectra of commercial Lupamin 9095 and purified PVAm with different degree of purification.

Lupamin 9095 and purified PVAm films (PVAm-LG and PVAm-HG) were analyzed by IR spectroscopy to assess the efficiency of the purification process; for completeness, the intermediate purification step post Soxhlet treatment (PVAm-LG+) was analyzed as well (Figure 5). The latter step did not cause massive variation of impurities concentration but was shown to greatly improve the efficiency of final ion exchange and was therefore maintained in the final purification procedure.

The peaks at 770, 1350, 1570, 2725 and 2840 cm^−1^ refer to the presence of the sodium formate salt (by comparison with the NIST’s IR spectra [64]) [65]. They are well visible in the commercial Lupamin 9095 spectrum but lose intensity in the purified PVAm spectra. In particular, PVAm-LG and PVAm-LG+ show depressed absorbance at the mentioned wavelengths with respect to Lupamin 9095, but the peaks are still present, suggesting a significant but not complete purification. In PVAm-HG films, on the other hand, the characteristic signals of the salt are almost completely absent (except for the one at 770 cm^−1^), indicating an excellent purification of the polymer. It is worth noticing that an intensification of the characteristic polymer signals (880 cm^−1^ N–H wagging, 1380 and 1440 cm^−1^ C–H bending/rocking, 1660 cm^−1^ N–H bending, 2920 cm^−1^ C–H stretching, and 3150–3400 cm^−1^ broad peak N–H stretching) is also observed in these materials, which corresponds to a better degree of purification.

Concerning the polymer peaks, then, an interesting change in the different spectra is the shift of the peak related to N–H stretching before and after the resin treatment (PVAm-HG). This one moves from 3100 to 3300 cm^−1^, which is quite reasonably associated to the neutralization of amine group from the R–NH_3_^+^ to the R–NH_2_ form, as also confirmed by the results obtained by Annenkov et al. [66]. This neutralizing effect is caused by the hydroxylated resin, which exchanges hydroxyl anions in solution, while retaining salt anions.

#### 3.1.2. Thermal Characterization

**Figure 6:**
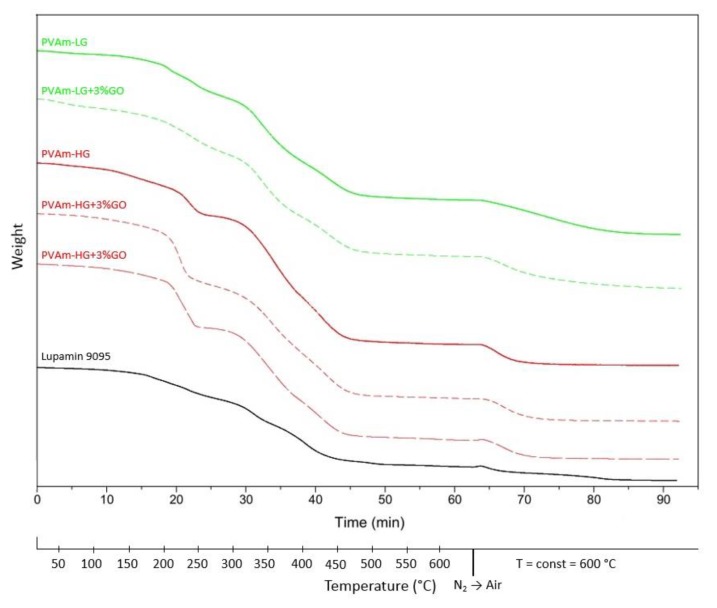
TGA curves of Lupamin 9095 and PVAm films at different degrees of purification, with and without G and GO.

Thermal and thermo-oxidative stability of Lupamin 9095 and purified PVAm was assessed through TGA analysis (Figure 6). The overall behavior of all tested samples is quite similar and the most important differences concern the entity of the weight loss (visible in Table 1) and final residues. 

Until 180 °C, TGA curves display weight loss due to absorbed water. The polymer degradation occurs in two main steps with onset temperature at 190–220 and 305–320 °C with only very minor differences among different materials, as visible in Table 1. During degradation, ammonia and hydrazine are released by the polymer, as stated by IR spectra of the evolved gas.

Sample weight is stable in the range 500–600 °C, indicating the end of the thermal degradation. Organic residues were oxidized by switching in air atmosphere at 600 °C and keeping the sample at that temperature for 30 min. After this treatment, purified films showed a significantly lower residue with respect to untreated Lupamin 9095. In particular, PVAm-LG and PVAm-HG showed residues in the order of 15% and 8% of the initial weight, respectively, confirming the better purification obtained when the ion exchange step was used after ethanol washing.

No major modifications of the TGA curves of the polymer were observed upon graphene or graphene oxide addition; these nanofiller, therefore, did not seem to affect the thermo-oxydative stability of the matrix.

**Figure 7:**
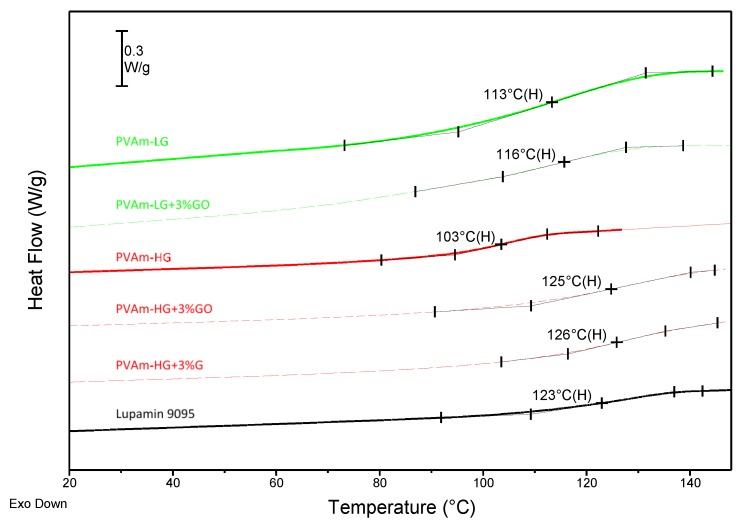
DSC curves of Lupamin 9095 and PVAm films at different degrees of purification, with and without G and GO. Temperatures in the chart refer to T_g_, which was calculated considering the maximum value of lines’ slopes, during the second order transition.

Interestingly, the degree of purification and nanofiller loading affected the glass transition temperature (T_g_) of the films, as shown by DSC analyses reported in Figure 7.

In general, PVAm T_g_ decreases when purification is improved, a trend that is clearly related with the salt content, which seems to make the overall system more rigid. For similar reasons, the presence of nanofillers tends to increase the T_g_ in a different way, depending on the material considered. The determined glass transition temperatures are shown in Figure 7 and summarized in Table 1.

More specifically, in the case of Lupamin9095, the very high salt content creates, upon film deposition, macroscopic heterogeneous agglomerates, which are clearly visible to the naked eye. This material therefore presents a two-phase system, in which the T_g_ is associated to the polymeric phase. 

Lightly purified PVAm, on the other hand, showed very small crystals with micrometric dimensions (see SEM analysis below), which seem to act as a nanometric filler able to efficiently rigidify and reinforce the matrix through polar interactions with the polymeric chains. This hypothesis can explain the higher T_g_ with respect to the PVAm-HG and the very small effect of GO addition, which indeed causes only a 3 °C increase on the Tg in PVAm-LG + 3% GO with respect to PVAm-LG. The presence of micrometric sodium formate inclusions in low purified Lupamin indeed can interact and attract the polar GO inside to the compatible ionic structure of such salt, thus keeping unmodified the macromolecular rigidity.

On the other hand, 3% in weight addition of both G and GO to PVAm-HG determined a sharp increase of T_g_ in High Grade films (+20 °C), as in this case no rigid inclusions are present in the highly purified polymer, thus the nanofiller can properly act as reinforcement, hindering macromolecular motion.

#### 3.1.3. SEM Results

Through SEM analysis, the fractured surfaces of produced films were examined to investigate the dispersion and the interaction of G and GO with the polymeric matrix.

**Figure 8:**
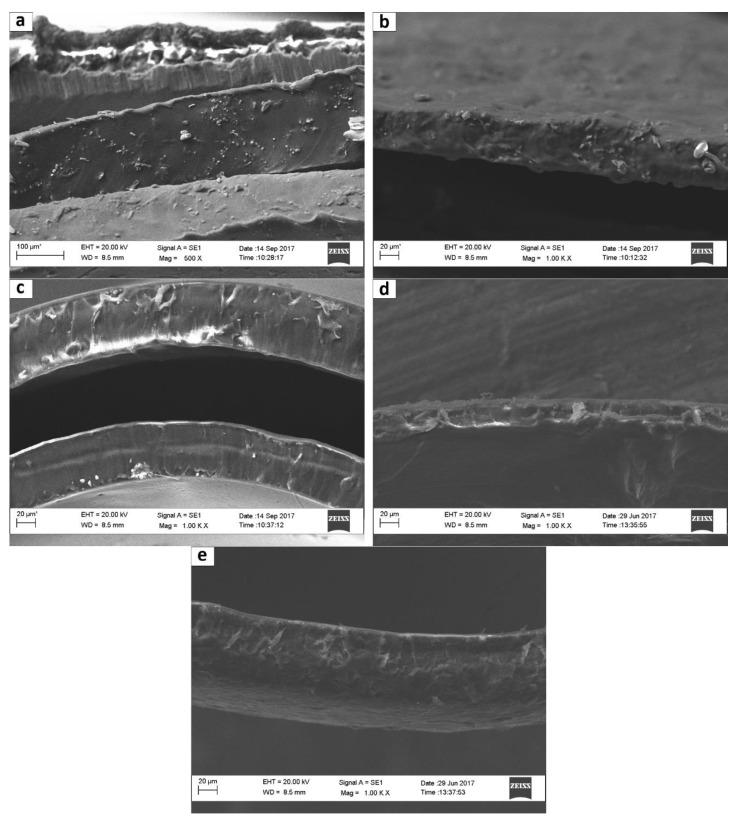
SEM images of the fracture surfaces of: (**a**) PVAm-LG; (**b**) PVAm-LG + 3% GO; (**c**) PVAm-HG; (**d**) PVAm-HG + 3% GO; and (**e**) PVAm-HG + 3% G.

As shown in Figure 8, the fracture surface of the PVAm-HG film (Figure 8c) is much smoother than the PVAm-LG (Figure 8a), most likely due to the presence of a considerable amount of sodium formate in the low-grade film, still visible also in PVAm-LG + 3%GO film (Figure 8b). The addition of G and GO in the high-grade film (Figure 8d,e), on the other hand, does not appears to modify the structure of the materials, even though the presence of a slightly higher heterogeneity could be argued, possibly due to the nano-reinforcement presence.

Differences in smoothness between the fracture surfaces may be considered as further proof of the achieved purification: the most purified materials presented fewer salt impurities on the surface.

#### 3.1.4. Water Sorption Results

**Figure 9:**
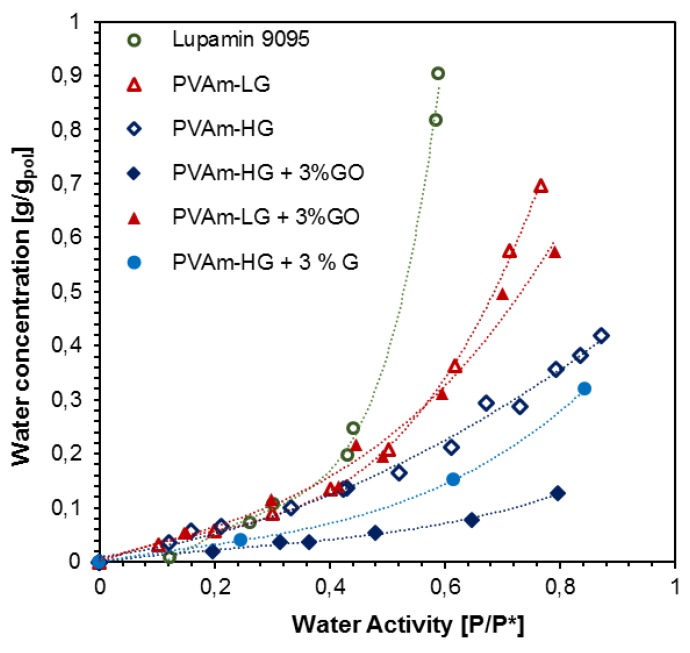
Water sorption isotherms at 35 °C for PVAm at different degrees of purification and with different types of filler. Lupamin 9095 sorption data from [40].

Figure 9 shows the water sorption isotherms obtained for the various materials at a temperature of 35 °C.

In the chart, it can be seen how Lupamin 9095, the commercial form, can absorb the largest amount of water, reaching 90 wt% of water mass gained at 60% water activity, as also shown in a previous work [40]. It is followed by PVAm-LG (60–70 wt% increase at 75–80% water activity), and PVAm-HG (10–40% mass gained at 80–90% water activity). As expected, the removal of a hygroscopic phase such as the saline one, largely reduced the water uptake and the matrix swelling.

The addition of a graphene-based phase presents different effects on the water sorption of the different materials. Indeed, the reduction of water uptake upon filler loading is negligible for PVAm-LG composites, while it results significantly higher for PVAm-HG based materials. 

In the latter case, even at relatively small amounts, the graphene-based nanofillers significantly decreases the water uptake of PVAm-HG based composites with respect to that of the non-loaded corresponding polymer. For example, pure PVAm-HG presents an uptake of 21 wt% at around 60% activity, while the addition of 3 wt% graphene and 3 wt% graphene oxide lower that value to, respectively, 15.1 and 7.8 wt%. This decrement can be interpreted as a positive interaction between the matrix and the filler, which is capable of stiffening the overall material structure, preventing an excessive swelling at high relative humidity. This result is more evident in the case of GO rather than G, likely due to the larger aspect ratio of the sheets and to the possible presence of electrostatic interaction among the carboxylic groups of GO and the primary amine groups of PVAm.

Concerning the PVAm-LG + 3% GO behavior, it can be noted instead that it is in accordance with that discussed above regarding the materials glass transition temperature variation: the limited effect of the filler seems indeed to be related to the presence of finer dispersion of sodium formate crystals, which somewhat interact with the GO decreasing its ability to impact the properties of the polymeric matrix, and also to the cationic nature of low-grade samples, which impairs the possible interactions among the amine groups of the polymer and the GO’s carboxylic acid groups.

### 3.2. Permeation Results

**Figure 10:**
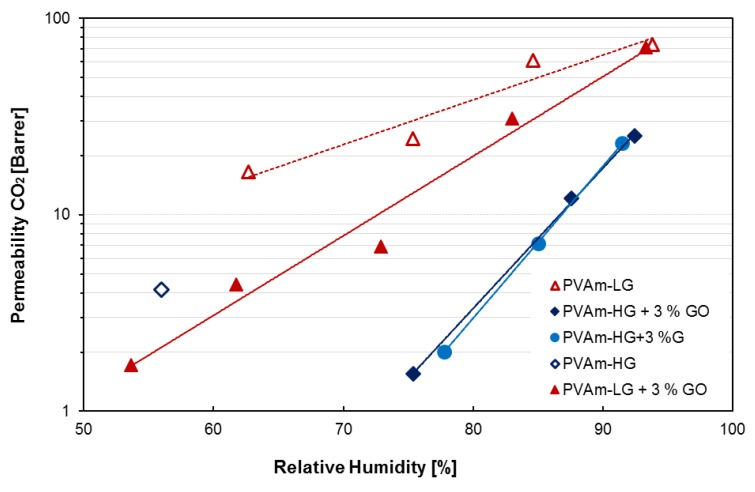
Single gas CO_2_ permeability of PVAm based films with respect to relative humidity, with trend lines**.**

The single gas permeation results for carbon dioxide for the different membranes tested are presented in Figure 10 as a function of relative humidity and for further clarity in Table 2, where CO_2_/N_2_ selectivity are also reported, when available. As quite common for hydrophilic membranes, permeability increased exponentially with the degree of humidification [67] due to the high water sorption and consecutive membrane swelling.

Apart from the general behavior and considering the different materials, as a first observation, it can be noted that for PVAm-HG a single point at intermediate humidity was obtained. This is due to the fact that PVAm in its pure form appeared to be quite unstable as a self-standing film, especially when purified. This led to the impossibility of running tests at higher humidity, due to the rupture of the film, verified on several specimens. For the case of PVAm-LG, the presence of residual sodium formate appeared to slightly increase the mechanical stability, allowing a full permeation curve to be acquired, at least for CO_2_; in the case of nitrogen tests, no reliable data could be obtained, likely due to the longer experimental time required, which resulted in an excessive stress on the film. On the other hand, all films prepared using a graphene-based filler resulted mechanically stable and allowed extensive tests to be performed without any loss in permselective properties for several days. 

From the experimental data, it can be seen how PVAm-HG presents a permeability of 4.2 Barrer at 56% RH, but no particular trend can be obviously inferred. For PVAm-LG, permeability of CO_2_ varies from 16.5 Barrer at 63% RH, up to 73.8 Barrer at 93% RH. For the same polymer, filled with 3 wt% of graphene oxide, values range from 1.7 Barrer at 53% RH to 71.0 Barrer at high humidity. PVAm-HG, loaded with the same quantity of graphene oxide, presented instead a permeability for carbon dioxide of 1.6 and 25.1 Barrer at 75% and 93% RH, respectively. When loaded with few-layer graphene, the same matrix presented values from 2.0 Barrer at 77% RH to 23.1 Barrer at 92% RH. 

The non-loaded materials therefore present a higher permeability at the same relative humidity with respect to their counterparts containing a graphene-based nanophase. This is a somewhat expected result, since the platelets of graphene, due to their high aspect ratio, can easily increase the tortuosity of the diffusion pathway of the gases in the matrix. This effect appeared to be less pronounced in the case of PVAm-LG, possibly due to the polar interaction between graphene oxide and salt nanocrystal in these materials as already discussed by considering DSC and water sorption experimental results.

Regarding PVAm-HG, it is quite interesting to notice how no particular differences in gas permeability can be discerned between the materials obtained using two different fillers. This could be contradictory considering that GO usually presents a higher aspect ratio than few-layers graphene and that PVAm-HG + GO showed a lower water uptake than PVAm-HG + G. Usually, indeed, when no other factors are in play, a lower concentration of humidity in the matrix resulted in a lower CO_2_ permeability, as also shown by previous works [26,27,32,37,39,40]. The fact that the two composites show comparable permeabilities therefore suggests the existence of a different structure and possibly of higher interaction of carbon dioxide with the polarly charged GO, which increase the intrinsic materials permeability thus compensating negative factor mentioned above.

**Figure 11:**
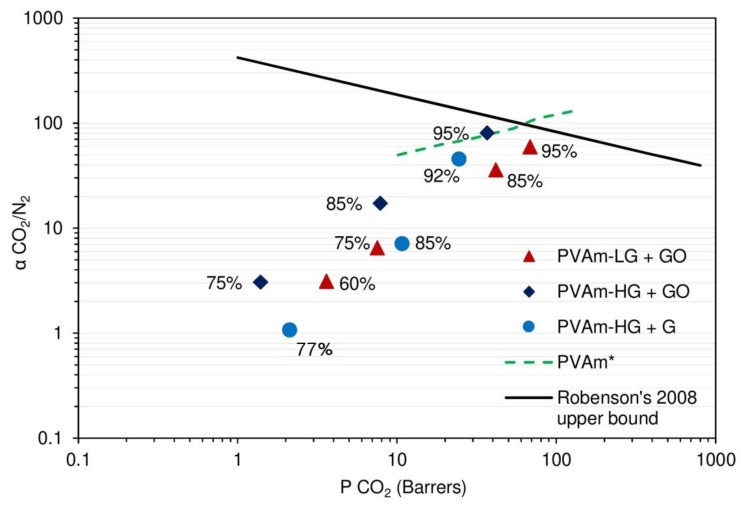
CO_2_/N_2_ selectivity of graphene loaded films on a Robeson plot. PVAm* data refer to pure PVAm permeability as obtained in Ref. [43]**.**

As previously mentioned, nitrogen permeation tests were only possible on the nanocomposite membranes, due to their enhanced stability. For this reason, the ideal selectivity could be calculated only for these materials, using an exponential interpolation to report data from different gases at the same RH values. Figure 11 presents the results from this calculation in a Robeson plot. It can be seen how for all materials a monotonous trend of selectivity versus permeability is followed, with both quantities increasing with relative humidity, indicated next to each experimental point for reader convenience. These behaviors were observed in previous studies [32,33] and were somewhat expected because water favors both CO_2_ solubilization and facilitated transport execution, as previously shown in Equations (1)–(5) and Figure 1, thus increasing its permeability more than that of nitrogen.

In the case of PVAm-LG+GO in particular, selectivity was shown to vary from 3.1 to 59.2 when humidity was raised from 60% to 95%. For PVAm-HG, instead, this value ranges from 3.0 at 75% RH to 80.7 at 95% RH for the graphene oxide sample and from 1.1 at 82% RH to 45.2 at 92% RH for the graphene one.

Analyzing PVAm-LG and -HG reinforced with graphene oxide, therefore, it resulted that the low-grade polymer has higher maximum CO_2_ permeability (≈70 Barrer), with lower CO_2_/N_2_ selectivity (≈60) compared to the high-grade one, which has higher selectivity (≈80) and lower permeability (≈35 Barrer). These differences are probably due to the swelling difference of the two composite materials, as already discussed, and shows a PVAm-LG + 3% GO more hydrophilic than the PVAm-HG-based composite. These results fit also with the chemical structure differences between the two materials: PVAm-HG has an almost totally neutral charge upon the polymeric chain, leading to a tighter packing of the chain itself, which cause the overall lower permeability, while the PVAm-LG material has much higher ionic repulsion [56]. Nevertheless, the higher neutral secondary amine group concentration in PVAm-HG promotes the facilitated transport mechanism shown in Figure 1 and Equations (1)–(5), which results in higher CO_2_/N_2_ selectivity.

Despite these differences, the two materials show a very similar behavior in terms of overall permselective performances as they lie very close, but are not able to overcome the CO_2_/N_2_ Robeson’s upper bound [16], even at the highest humidity inspected.

From this point of view, therefore, contrary to what expected, the further purification step seems not to give substantial advantages in terms of membrane performance. 

Among the many papers found in the literature about PVAm based system, it is very difficult to find references for a consistent comparison of the present results in order to give a more general picture of the overall potential of such membranes. Available data on FTM are usually measured at higher temperatures and/or different upstream pressures, and often refer to very complex systems also including mobile carriers. Among others, Zhao and coworkers, studying PVAm-based mixed-matrix membranes, tested PVAm thin films at 22 °C and 100% RH and reported CO_2_ permeabilities in the order of 50 Barrer and CO_2_/N_2_ selectivity lower than 10 [68]. On the other hand, 260 Barrer and a selectivity of 24 were found by Hamouda et al. when testing at the same temperature and pressure (1 bar and 25 °C) a polyetherimine/polyvinyl alcohol/polyethyleneglycol polymer blend [31], at 20% RH; in the former case, lower selectivity was achieved with similar permeability, while, in the latter, higher permeability but lower selectivity was obtained with respect to the here considered membranes. A more interesting comparison in this concern is given by the data for a supported purified PVAm membranes reported by Kim et al. [43]**,** as also reported in Figure 11. In this case, values are generally higher than those obtained in the present work and confirm that graphene addition mainly affects polymer permeability, while it has a limited effect on selectivity.

This reduction of the separation performance with respect to the supported pure polymer from Kim et al. [43]**,** however, is accompanied by a remarkable improvement of mechanical strength of the material: pristine PVAm sample indeed cannot be tested as pure polymer self-standing membrane and also literature data [25] were obtained from thin supported films; the present composites instead were tested routinely with no problem for more than a month without showing any rupture or degradation.

## 4. Conclusions

Graphene-based PVAm composite membranes were fabricated and tested to assess their potential in the field of CO_2_ capture and purification. 

The materials were produced by mixing polyvinylamine (PVAm) at different degrees of purification with few-layer graphene and graphene oxide, in an attempt to increase the mechanical stability of the water-soluble polymer without excessively reducing its transport properties.

Commercial PVAm, Lupamin 9095, was purified via a multistep procedure to obtain two grades of pure materials, PVAm-LG and PVAm-HG, which have, respectively, low and high purification grade. These samples were then loaded with 3 wt% of few-layer graphene or graphene oxide and were characterized through FTIR, SEM, TGA, and DSC techniques as well as by measuring water vapor sorption and CO_2_ and N_2_ permeability at different relative humidity.

The results obtained confirm the sodium formate removal during the purification steps and also the neutralization of amine groups on the polymeric chain in the purest product (PVAm-HG), which should improve the facilitated transport performance of the membrane. In addition, PVAm-HG also seems to be able to better disperse the nanofiller with respect to PVAm-LG; both DSC and water sorption results were greatly affected by the addition of the nanofiller in the highly purified polymer while remained substantially unchanged in the partially purified material. SEM images in this concern showed in the PVAm-LG materials the presence of sodium formate nanocrystal, which may interact with the polar graphenic nanofiller affecting its dispersion in the matrix. 

Permeation tests, conducted at 35 °C, gave some interesting results at high relative humidity (95% RH), with a maximum CO_2_ permeability of almost 70 Barrer (PVAm-LG + 3% GO) and maximum CO_2_/N_2_ selectivity of about 81 (PVAm-HG + 3% GO). In general, both permeability and selectivity increased with the increasing of relative humidity and water content in the polymer. Moreover, PVAm-HG resulted to be more selective and less permeable than PVAm-LG-based materials and, among different nanofiller GO, resulted to give similar permeability but slightly higher selectivity with respect to graphene. 

Therefore, the further purification step did not seem to remarkably modify the materials separation performances, which in any case resulted to be below the Robeson’s upper bound, for all the materials considered. This is likely because of the barrier effect performed by the high quantity of filler added to the system. Graphene-based nano-sheets, however, allowed achieving complete permeation tests on self-standing membranes, which was not possible in polymer without filler, due to film rupture at high relative humidity. In this way, graphene addition has confirmed the ability to act as a reinforcer for this type of polymers containing in side-chain amino groups, thus presenting some potential as an additive for FTM membranes applications. Further research, however, has to be performed to prevent excessive reduction of the pristine PVAm separation performances upon nanofiller addition.

## Figures and Tables

**Table 1:** Onset degradation temperatures, weight loss (from TGA) and glass transition temperature (T_g_) (from DSC) of commercial Lupamin 9095 and purified PVAm with and without G and GO.

Sample	First Degradation Step		Second Degradation Step		T_g_ (°C)
	Onset T (°C)	Weight Loss (%)	Onset T (°C)	Weight Loss (%)	
Lupamin 9095	190	~14	305	~32	123
PVAm-LG	190	~21	315	~48	113
PVAm-LG + 3% GO	195	~25	320	~48	116
PVAm-HG	210	~24	315	~60	103
PVAm-HG + 3% GO	220	~21	315	~65	125
PVAm-HG + 3% G	210	~30	310	~51	126

**Table 2:** Permeselective properties of the different membranes PVAm membranes.

Membranes	RH %	CO_2_ Permeability (Barrer)	CO_2_/N_2_ Selectivity
PVAm-Hg	56	4.2	n.d.
PVAm-LG	63	16.5	n.d.
	75	24.4	n.d.
	85	61.2	n.d.
	93	73.8	n.d.
PVAm-LG + 3% GO	53	1.7	0.1
	62	4.4	3.1
	73	6.9	6.5
	83	31.0	36.1
	93	71.0	59.0
PVAm-HG + 3% GO	75	1.6	3.0
	87	12.0	17.4
	93	25.1	80.6
PVAm-HG + 3% G	77	2.0	1.1
	85	7.1	7.04
	92	23.1	45.2
